# First person – Hamna Ammar

**DOI:** 10.1242/bio.062595

**Published:** 2026-04-22

## Abstract

First Person is a series of interviews with the first authors of a selection of papers published in Biology Open, helping researchers promote themselves alongside their papers. Hamna Ammar is first author on ‘ Enhancer of zeste homolog 2 (Ezh2) is required in mouse Isl1-expressing progenitors for proper development of cardiac and skeletal hindlimb structures’, published in BiO. Hamna conducted the research described in this article while an undergraduate student researcher in Dr Paul Delgado-Olguin's lab at the Peter Gilgan Center of Research and Learning, The Hospital for Sick Children, Toronto, Canada. She is now an MSc graduate student in the lab of Dr Lena Serghides at The University of Toronto, investigating genetic and environmental determinants of cardiovascular diseases, with a special focus on maternal cardiovascular health and congenital cardiovascular disorders.

**Figure:**
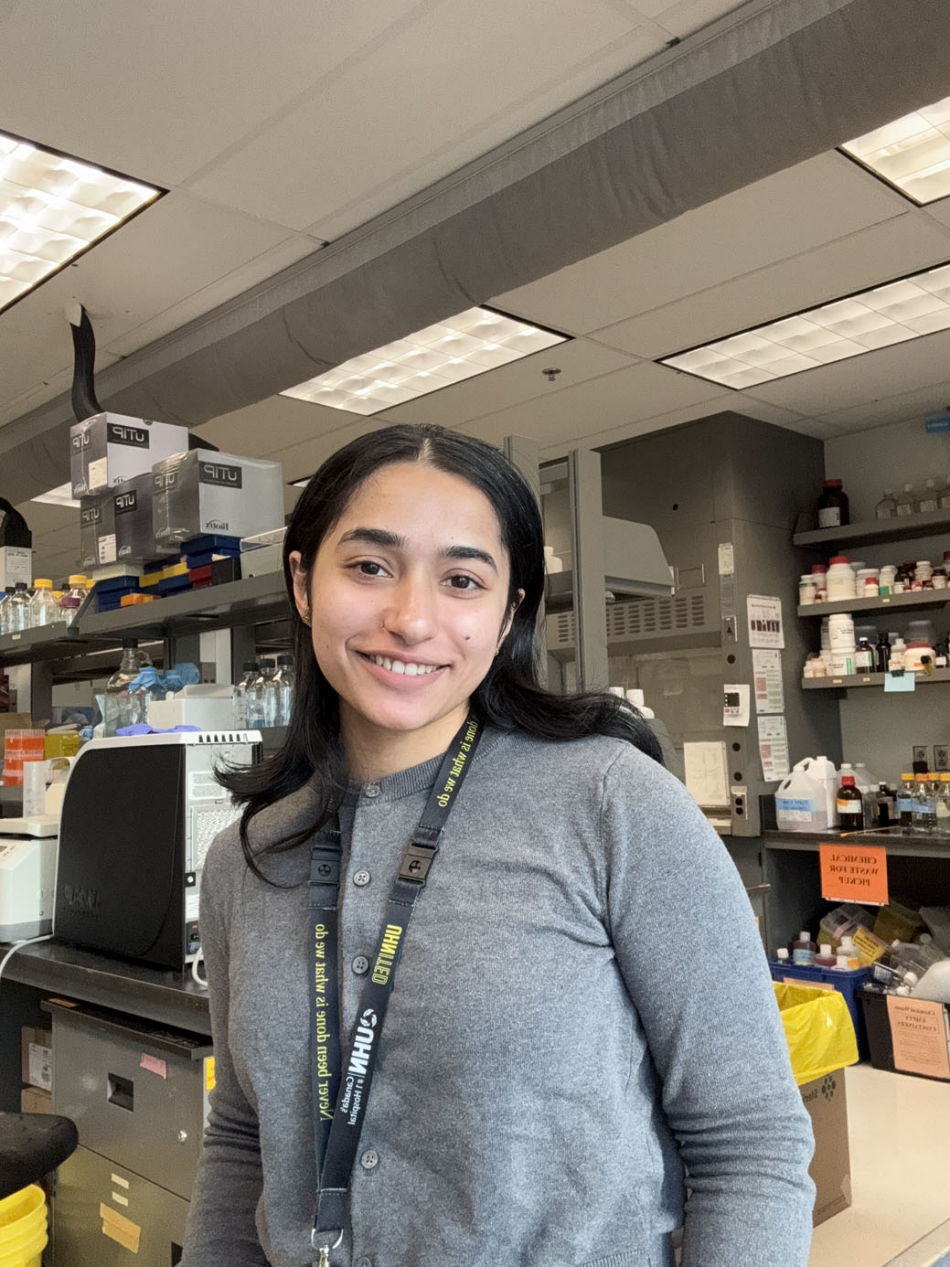
Hamna Ammar

**Describe your scientific journey and your current research focus**

My research journey began as an undergraduate student in the Health and Disease program at the University of Toronto, where I was first introduced to experimental research through an intensive laboratory course in plant molecular biology. During this course, I had the opportunity to learn and apply molecular techniques such as using CRISPR/Cas9 to knock out a protein essential for chloroplast synthesis. This experience sparked a strong interest in molecular biology and led me to spend a full summer in a plant lab further developing my technical skills. As I became more confident in my skills, I also began to develop a strong interest in understanding the molecular mechanisms behind human disease, leading me to pursue an undergraduate project in the Delgado lab at the SickKids Research Institute, Canada, investigating the role of epigenetic regulators in fetal cardiovascular development. Through this work, I developed an appreciation for how transcriptional and epigenetic regulation drive developmental gene networks and how their disruption can contribute to disease. Working with pregnant mice over two years also sparked a special interest in maternal cardiovascular health and its influence on fetal outcomes. This led me to my current position in the Serghides lab at where I am now focused on investigating maternal cardiovascular outcomes in HIV-infected mice treated with antiretroviral therapy.

**Who or what inspired you to become a scientist?**

My inspiration to become a scientist and pursue research came from the great mentors and the people I've met along the way. I think it all started with a teaching assistant in my first plant molecular biology course who helped me appreciate the science behind all the experimental techniques we used in the lab, who showed me and made me appreciate how every molecular reaction we set up is like a puzzle piece contributing to a larger question. I was also very fortunate to conduct my undergraduate research under Dr Delgado-Olguín, who encouraged curiosity and critical thinking while constantly challenging my understanding of the work I was doing. He treated me as a fellow scientist and taught me to take responsibility for the quality of my work. Lastly, I drew inspiration from my lab mates and all the thought-provoking discussions we had about both their research and my own which made the process feel exciting and intellectually rewarding. It was incredibly motivating to be part of a community that shares a genuine enthusiasm for discovery and the advancement of knowledge. Throughout my research, I have been surrounded by people who view a strong research question as an enticing challenge and each experiment as a step closer to an answer.

**How would you explain the main finding of your paper?**

My research found that removing a gene that has different roles in development leads to very specific defects in the heart and bones of mice.

**What are the potential implications of this finding for your field of research?**

Our findings elucidate the importance of *Ezh2*, an epigenetic factor important for the regulation of gene activity during early development, in coordinating heart and limb development. This finding improves our understanding of the underlying causes of congenital diseases that show multisystemic defects such as those in the heart and limbs.

**Figure:**
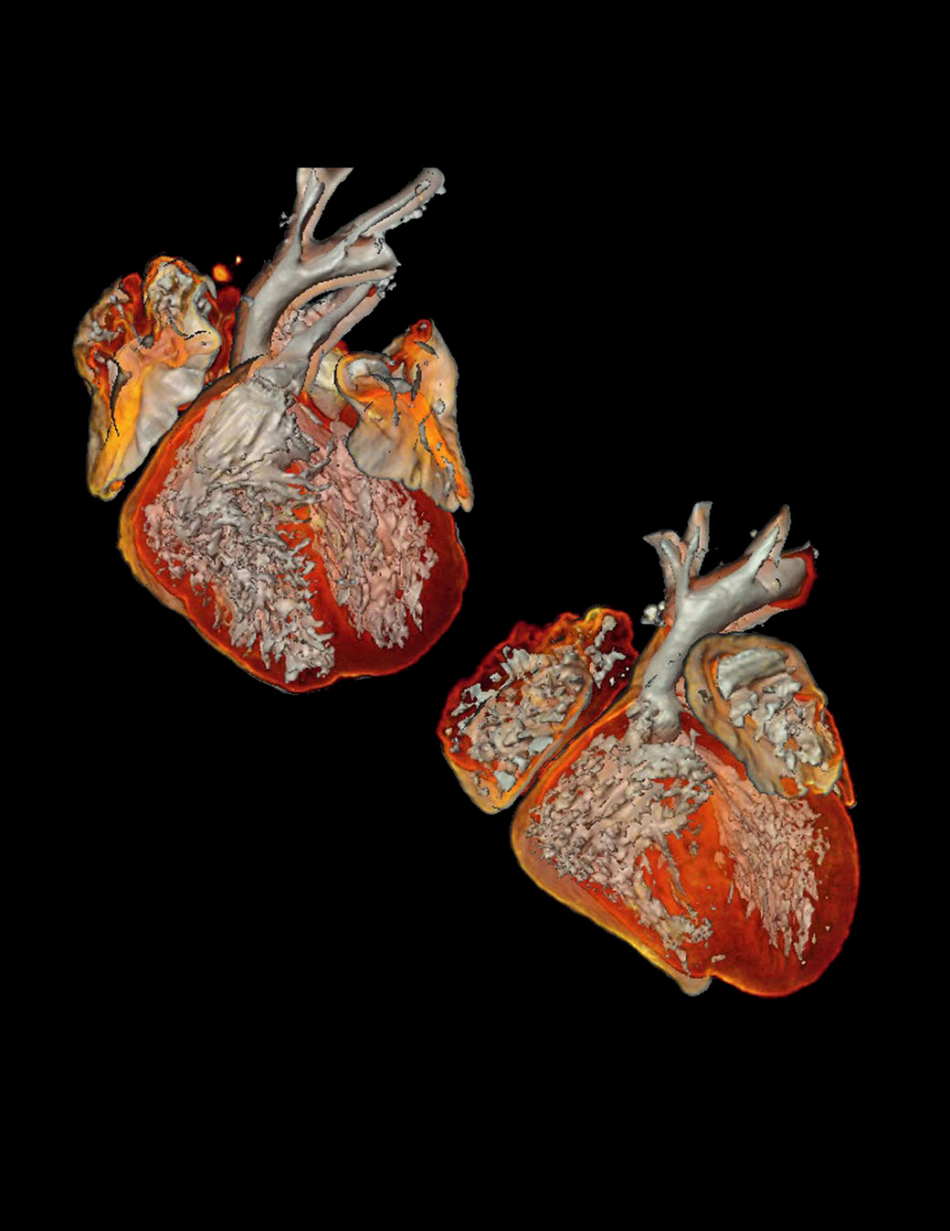
Optical projection tomography (OPT) image showing presence of a persistent truncus arteriosus, an outflow tract defect, in mouse embryos on gestational day 18.5.

**Which part of this research project was the most rewarding?**

The most rewarding and the most memorable part of this project was most definitely when I cut the first set of sections that revealed evidence of an outflow tract defect in the heart. It was the first real indication that we were onto something, and it set the direction for the rest of the study. The technique was completely new to me; it took me days of practice and hours of careful, meticulous work to get the sectioning right. But when I saw those sections for the first time, it made all of the effort and hard work feel worthwhile. I still remember the moment clearly when I first looked at the slide and thought: “I think I found it.”.

The most rewarding and the most memorable part of this project was most definitely when I cut the first set of sections that revealed evidence of an outflow tract defect in the heart

**What do you enjoy most about being an early-career researcher?**

The most fun part about being an early-career researcher for me, in all honesty, is the sense of accomplishment that you feel after learning and mastering a new technique. I find it rewarding working and your progress over time, putting in the work, and then realising afterwards that you can now do something that felt completely unfamiliar just a few weeks ago.

**What piece of advice would you give to the next generation of researchers?**

My advice to the next generation of researchers is to try, and fail. Keep trying and keep failing because somewhere between trying and failing, you'll find success, meaningful friendships, and lots of resilience.

**What's next for you?**

I plan to continue pursuing research that helps deepen our understanding of developmental biology and cardiovascular disease. My focus now is improving our knowledge of maternal cardiovascular health and pregnancy related outcomes in the context of HIV viral infection and antiretroviral treatment. I am especially interested in translating discoveries from animal models into clinically relevant insights that can inform important decisions about human disease.
